# Substrate Mapping and Ablation for Ventricular Tachycardia in Patients with Structural Heart Disease: How to Identify Ventricular Tachycardia Substrate

**DOI:** 10.19102/icrm.2019.100302

**Published:** 2019-03-15

**Authors:** Takeshi Kitamura, Claire A. Martin, Konstantinos Vlachos, Ruairidh Martin, Antonio Frontera, Masateru Takigawa, Nathaniel Thompson, Ghassen Cheniti, Gregoire Massouille, Anna Lam, Felix Bourier, Josselin Duchateau, Thomas Pambrun, Arnaud Denis, Nicolas Derval, Meleze Hocini, Michel HaÏssaguerre, Hubert Cochet, Pierre JaÏs, Frédéric Sacher

**Affiliations:** ^1^IHU Liryc, Electrophysiology and Heart Modeling Institute, Fondation Bordeaux Université, Pessac-Bordeaux, France; ^2^Electrophysiology and Ablation Unit, Bordeaux University Hospital (CHU), Pessac, France; ^3^Centre de recherche Cardio-Thoracique de Bordeaux, University of Bordeaux, Bordeaux, France; ^4^Royal Papworth Hospital NHS Foundation Trust, Cambridge, UK; ^5^Newcastle University, Newcastle-upon-Tyne, UK; ^6^San Raffaele Hospital, Milan, Italy; ^7^Tokyo Metropolitan Hiroo Hospital, Tokyo, Japan

**Keywords:** Ablation, cardiac imaging, substrate, three-dimensional mapping system, ventricular tachycardia

## Abstract

Catheter ablation for ventricular tachycardia (VT) has been increasingly used over the past two decades in patients with structural heart disease (SHD). In these individuals, a substrate mapping strategy is being more commonly applied to identify targets for VT ablation, which has been shown to be more effective versus targeting mappable VTs alone. There are a number of substrate mapping methods in existence that aim to explore potential VT isthmuses, although their success rates vary. Most of the reported electrogram-based mapping studies have been performed with ablation catheters; meanwhile, the use of multipolar mapping catheters with smaller electrodes and closer interelectrode spacing has emerged, which allows for an assessment of detailed near-field abnormal electrograms at a higher resolution. Another recent advancement has occurred in the use of imaging techniques in VT ablation, particularly in refining the substrate. The goal of this paper is to review the key developments and limitations of current mapping strategies of substrate-based VT ablation and their outcomes. In addition, we briefly summarize the role of cardiac imaging in delineating VT substrate.

## Introduction

Over the last decade, catheter ablation for ventricular tachycardia (VT) has been increasingly performed as an adjunctive therapy to antiarrhythmic drugs.^1,2^ With this came an increased understanding of the mechanisms of VT; notably, it commonly results due to scar-related reentry in patients with structural heart disease (SHD). Activation mapping and entrainment mapping are reasonable approaches to identify and target critical sites of the reentrant VT circuit for ablation in patients with tolerated reentrant VT.^3–6^ However, the majority of patients presenting for catheter ablation have unstable VT that hampers the accurate definition of the critical areas of the reentrant circuit with activation or entrainment mapping.^7,8^

Thus, substrate ablation is increasingly favored as a VT ablation strategy, with or without entrainment/activation mapping methods. Substrate-based approaches involve the identification of local abnormal ventricular electrograms that represent diseased areas consistent with potential isthmuses capable of supporting reentrant VT and may be followed even when VTs are not inducible or not hemodynamically tolerated.^9,10^ Although a combination of several approaches is commonly employed during VT ablation, a number of studies have examined with variable results whether a substrate-based ablation strategy may be superior or comparable to one guided predominantly by the activation/entrainment mapping of inducible and hemodynamically tolerated VTs.^11–18^ Critical in achieving more successful results with a substrate-based approach is an accurate representation of the pathologic substrate; various strategies to delineate this have been proposed to date.^4,10,13–22^

With the advent of three-dimensional (3D) electroanatomical mapping (EAM) systems in the 1990s,^23^ there has been a significant improvement in our ability to represent both anatomical and functional electrical information in a real-time model of the ventricle. Furthermore, the development of multipolar catheters facilitates precise scar detection,^24–26^ with potentially more successful results.^27^ In addition, cardiac imaging in the form of cardiac magnetic resonance (CMR) imaging or multidetector computed tomography (MDCT) may play a potentially important role in the preprocedural assessment of cardiac anatomy and myocardial substrate and the intraprocedural integration of the structural and electrophysiological VT substrate.^28–31^

In this paper, we review substrate mapping and ablation strategies and clinical outcomes for VTs in the setting of SHD and also discuss the importance of modalities for substrate detection in addition to those based on electrograms.

## Substrate mapping in patients with structural heart disease

### Electrogram-based substrate detection

**Figure 1:**
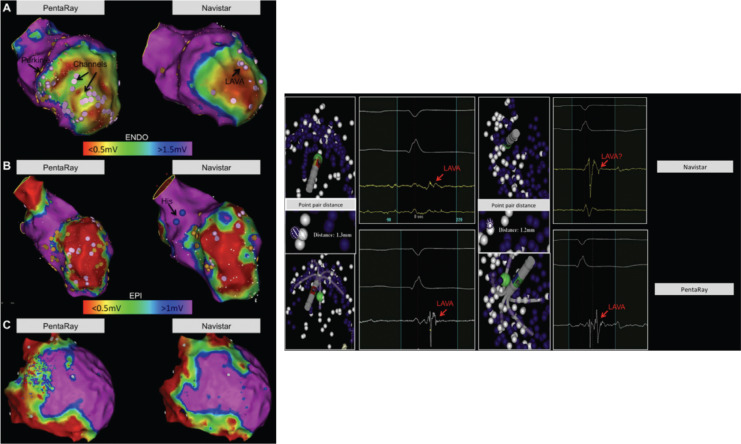
**Left:** Comparison of bipolar voltage maps (endocardial: scar < 0.5 mV, border zone 0.5–1.5 mV, and healthy tissue > 1.5 mV; epicardial: border zone 0.5–1 mV and healthy tissue ; 1 mV) using Navistar^®^ (Biosense Webster, Diamond Bar, CA, USA) (NAV) mapping versus PentaRay^®^ (Biosense Webster, Diamond Bar, CA, USA) (PR) mapping in the endocardium **(A)** and epicardium **(B)** of a sheep model with an iatrogenic-created anteroseptal scar and in humans **(C)** using the CARTO^®^ 3 system (Biosense Webster, Diamond Bar, CA, USA). All images are shown in an anteroposterior view. **A:** LAVA is represented in pink, the proximal conduction (left-sided His) system is in blue, and the Purkinje system is in yellow. Border zone areas and LAVA channels are visible within the scar with PR mapping, but none are visible with NAV mapping. **B:** Three LAVA channels are visible with PR mapping but none with NAV mapping. The border zone is smaller and demonstrates increased detail using PR mapping. **C:** Larger scar area and improved border zone definition using PR versus NAV mapping. **Right:** Point pair analysis (≤ 3 mm of distance between a PR and NAV point) from two examples by manual signal analysis in two different patients. Substrate maps are shown at 100% transparence. LAVA using PR (in purple) and NAV (in white) are tagged. The distance between tags is measured using the distance measurement tool in the mapping system. A red arrow indicates a clear LAVA visible with PR mapping but one that is barely or not visible with NAV mapping. Reproduced with permission from: Berte B, Relan J, Sacher F, et al. Impact of electrode type on mapping of scar-related VT. *J Cardiovasc Electrophysiol.* 2015;26(11):1213–1223. LAVA: local abnormal ventricular activity; ENDO: endocardial; EPI: epicardial.

The identification and modification of the arrhythmogenic substrate in the endocardium and/or epicardium are increasingly considered as composing a primary ablation strategy in patients with SHD. This technique was originally developed in the absence of 3D mapping^9,32–34^; however, the development of 3D mapping systems in the late 1990s^23^ accelerated the use of electrogram-based techniques in detecting and localizing substrate reproducibly and feasibly. Furthermore, the recent use of ultra-high-density mapping with catheters with multiple small electrodes and closer interelectrode spacing has enhanced the speed, density, resolution, and detailed near-field signal assessment of mapping acquisition, reducing interpolation and possibly improving clinical outcomes^25–27,35–38^ **(Figure 1)**.

**Figure 2:**
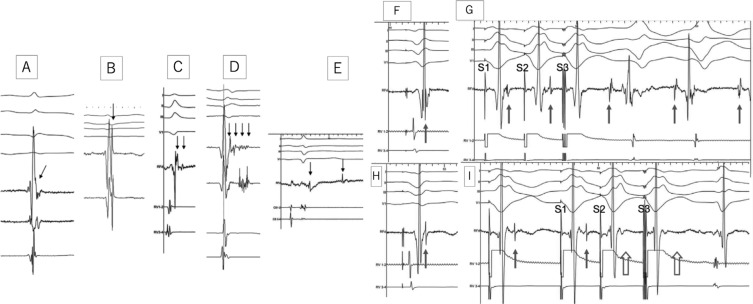
**Left:** Electrogram recordings from different patients showing characteristics of LAVAs (arrows). **A:** The potential representing LAVA is fused with the terminal portion of the far-field ventricular signal, making it difficult to identify the LAVA as a separate signal. **B:** The LAVA potential occurs just after and with a slightly higher frequency than the far-field ventricular potential. LAVAs in **A** and **B** occur within the QRS complex. **C:** The LAVA is a double-component potential that closely follows the far-field ventricular signal. The early component is a high-frequency potential that is almost fused with the preceding far-field ventricular potential. It occurs within the terminal portion of the QRS complex. Another low-amplitude signal follows an isoelectric interval and represents the late component of the LAVA, which occurs after the QRS complex. **D:** LAVAs are represented by pluricomponent signals without isoelectric intervals. These signals can be visualized distinctly from the preceding far-field ventricular signal. **E:** A double-component LAVA signal. Although the early component is recorded just after the QRS complex, the late component is recorded after the inscription of the T-wave on the surface electrocardiogram. **Right:** Role of LAVAs in the induction of VT and the influence of radiofrequency (RF) energy on LAVAs. **F:** The local ventricular electrogram during the baseline paced rhythm at first sight looks simple. However, in the terminal portion, a very high-frequency component (LAVA) may be identified. **G:** Programmed electric stimulation from the right ventricle (RV) unmasks the LAVA potential by increasing the delay from the far-field signal. The delay observed during RV pacing suggests poor coupling of the muscle bundle generating the LAVA signal. The delay is maximal with S3, which is associated not only with a change in the polarity of the LAVA but also with the induction of VT. **H:** After delivery of RF energy, there is a remarkable delay (see **A**) between the far-field ventricular signal and LAVAs during baseline paced rhythm. **I:** Repeat programmed electric stimulation from the RV results in the absence of LAVA signals after the far-field ventricular potential during S2 and S3 (open arrows). The absence of LAVAs is associated with an inability to induce VT. Although ablation has rendered the VT noninducible, further RF energy application is indicated to completely eliminate the LAVAs. Reproduced with permission from Jaïs P, Maury P, Khairy P, et al. Elimination of local abnormal ventricular activities: a new end point for substrate modification in patients with scar-related ventricular tachycardia. *Circulation.* 2012;125(18):2184–2196.

Bipolar voltage mapping to evaluate the electrogram peak-to-peak voltage is a widely accepted and frequently used technique to characterize substrate. Endocardially, a bipolar voltage amplitude of 1.5 mV or more normally identifies healthy tissue. Additionally, normal ventricular myocardial bipolar electrograms are defined as sharp, biphasic or triphasic signals with a duration of 70 ms or less and/or with an amplitude-to-duration ratio of more than 0.046.^9,39^ Areas with voltages of 0.5 mV to 1.5 mV are often considered as border zones in the setting of a 3.5-mm to 4-mm-tip, 1-mm ring electrode, and 2-mm interelectrode spacing mapping catheter **(Table 1)**,^10,32,34^ even in the right ventricle.^40^ The definitions above have been validated by human pathologic data and radiologic studies.^41–43^ Areas with voltages of less than 0.5 mV are generally considered as “dense scar”; however, low-amplitude abnormal electrograms are frequently observed in these areas.^44^ Therefore, in order to define unexcitable scar, the area should contain no visible electrograms (ideally using mapping catheters with smaller and narrower-spaced bipolar electrodes) and have no local pacing capture.^45^ In the epicardium, a bipolar voltage cutoff of 1 mV or more^46^ or 1.5 mV^47^ is considered normal. With regard to the right ventricle, epicardial 1.5 mV can also be a reasonable bipolar voltage cutoff.^40^ The majority of VTs have critical circuits located in the scar border zone,^48^ which harbors abnormal electrograms^9,32,33^ [ie, fractionated potentials,^49^ double potentials, and late potentials (LPs), discrete and separated from the QRS by 40 ms^50^], which can be targeted by catheter ablation. However, it has been reported that a certain proportion of abnormal potentials are also located in regions with bipolar voltages of more than 1.5 mV,^19,21^ with abnormal electrograms occasionally unmasked by extrastimuli.^51,52^ We have recorded at least 3% of substrate defined as local abnormal ventricular activity (LAVA) in voltage zones of more than 1.5 mV (because of far-field signal annotation)^53^ **(Figures 2A–2D)**. Moreover, Tung et al.^54^ found that 18% of critical VT isthmuses were within low-voltage areas during pacing from the site but within normal amplitude (> 1.5 mV) areas with pacing from another site, indicating that voltage is affected by the activation wavefront.^54–56^ Therefore, a voltage map based on standard (0.5–1.5 mV) voltage criteria is not necessarily capable of delineating the entire possible VT substrate.

Furthermore, with the use of high-density mapping with small electrodes and narrower interelectrode spacing **(Figure 1)**, traditional definitions of substrate voltage need to be adjusted depending on the mapping catheter.^24,36^ The electric field recorded by a pair of electrodes on a novel mapping catheter is relatively small, recording precise local signals located just underneath the electrodes.^57,58^ Meanwhile, endocardial unipolar voltage mapping has a large field of view and is useful to identify septal, intramural, and/or epicardial substrate,^58–69^ with different amplitude thresholds present depending on the etiology of the disease. The use of bipolar mapping with small electrodes and closer interelectrode spacing in combination with unipolar mapping may constitute an optimal strategy.

In summary, there are several limitations^55^ of conventional voltage mapping for substrate detection, with variations occurring due to the wavefront of activation,^54–56^ catheter interelectrode spacing,^24,25^ interpolation,^63^ far-field peak annotation of multicomponent electrograms, catheter orientation^64^ and contact,^65,66^ and surrounding insulating tissue (eg, fat, edema). Conversely, nonelectrogram techniques of substrate detection such as cardiac imaging^29,30,67–69^ are unaffected by directions of wavefront activation or techniques based on specific electrogram characterizations (see later). Lastly, omnipolar mapping is a new development that may resolve some of these limitations by providing instantaneous catheter-tip wavefront direction and speed.^70,71^ With this mapping technology, local electrical field signals are determined and used to assess the traveling wavefront on a multielectrode catheter, rather than activation-based data acquisition, which may allow for a beat-to-beat determination of wave propagation information that is independent of electrode orientation or activation time. Further clinical validation of this technology is ongoing.

### Specific electrogram-based substrate ablation strategies

Substrate-based ablation approaches may differ between VT ablation centers. A number of methods are implemented during substrate modification, with a large variation in the amount of ablation energy delivered according to the preset mapping and endpoints of the procedure. The major strategies are summarized in **Table 1**. These studies must be cautiously interpreted because the mapping details or endpoints are often heterogeneous, even for the same strategy. In addition, although the advantage of multipolar mapping catheters has now been recognized, many previous studies have used ablation catheters for mapping.

#### Local abnormal ventricular activity–guided ablation

In a seminal study by our group, we reported a mapping and ablation strategy to homogenize substrate defined as LAVAs **(Figure 2**).^19–21^ Elimination of all LAVAs is associated with improved midterm and long-term arrhythmia-free survival.^19–21^ LAVAs are defined as sharp, high-frequency ventricular potentials, possibly of low amplitude, that are distinct from the far-field ventricular electrogram that occurs at any time during or after the far-field ventricular electrogram in sinus rhythm or before the far-field ventricular electrogram during VT, which sometimes display fractionation or double or multiple components separated by very-low-amplitude signals or an isoelectric interval and which are poorly coupled to the rest of the myocardium.^19,21^ Importantly, this strategy also targets abnormal substrate in so-called normal-voltage areas, although most LAVAs are generally observed in low-voltage areas.^53^ Clinical outcomes have been reported as including a 55% (88/159) VT freedom rate during a median follow-up of 47 months (range: 33–82 months) without antiarrhythmic drug therapy except for β-blockers.^20^

#### Linear ablation with cross-section of the scar and border-zone

Marchlinski et al.^10^ first described the use of linear ablation lesions to target multiple unmappable VTs. The technique involved the creation of contiguous lesions from the dense infarct area through the infarct border-zone and anchored to anatomic barriers or healthy myocardium. In their study,^10^ the ablation strategy resulted in a 75% (4/16) freedom from VT recurrence rate at the median follow-up point of eight months (range: 3–36 months). Additionally, Soejima et al.^72^ reported a VT freedom rate of 62.5% (25/40) at a mean follow-up point of 12 months ± six months in their study. In addition, this linear ablation approach was the main approach used in the Substrate Mapping and Ablation in Sinus Rhythm to Halt VT (SMASH VT) trial,^22^ a randomized study showing promising results.

#### Late-potential ablation

Definitions of LPs differ among studies.^16,73–78^ The initial description was of any electrogram with a duration extending beyond the end of the surface QRS.^32^ Modified definitions have subsequently been reported, often with an isoelectric line among multiple components in the bipolar signals.^73,74^ Regarding the clinical result, Arenal et al.^73^ first reported that after a mean follow-up of nine months ± four months, no VT recurrence was observed in 19 (79%) of 24 patients. Volkmer et al.^16^ additionally demonstrated a 71% VT freedom rate (7/25) in a follow-up period of 26 months ± 14 months. Nogami et al.^74^ reported a 67% (6/18) VT freedom rate over a relatively long follow-up period of 61 months ± 38 months in patients with ARVC. Garcia et al.^75^ and Bai et al.^76^ also demonstrated results of the elimination of delayed potentials or LPs in patients with ARVC with follow-up [VT freedom in 77% (10/13) patients during 18 months ± 13 months of follow-up and ventricular arrhythmia or implantable cardioverter-defibrillator (ICD) appropriate therapy freedom in 84.6% (22/26) during 39 months ± four months of follow-up, respectively]. More recently, Vergara et al.^77^ reported that, after a mean follow-up of 13 months ± four months, VT recurred in 10 patients (20%).

#### Scar homogenization

Di Biase et al.^17^ reported on a scar homogenization approach targeting all abnormal electrograms within low-voltage areas defined with conventional bipolar voltage criteria when mapping in sinus or paced rhythm. With this approach, abnormal electrograms are defined as any electrograms that have more than three deflections, an amplitude of less than 1.5 mV, and a duration of more than 70 ms. The acute ablation endpoint was either the elimination of abnormal electrograms or the loss of local capture at high-output pacing (20 mA output at a 10-ms pulse width). This approach can potentially eliminate more possible critical sites than more focused mapping approaches, but has limitations in patients with massive substrate, particularly under unstable conditions. During a mean follow-up of 25 months ± 10 months, the freedom from VT recurrence rate was 81% (35/43) in patients with ICM who showed scar homogenization.^17^ More recently, the results of a multicenter randomized study comparing scar homogenization with standard limited substrate ablation in patients with ICM were reported.^11^ At one year of follow-up, freedom from VT recurrence was achieved in 52% (31/60) of patients who underwent clinical VT ablation only versus in 85% (49/58) of patients who underwent scar homogenization.

#### Border-zone ablation/core isolation

The core isolation approach was recently developed by Tzou et al.^79^ in an effort to limit the number of lesions required to eliminate all of the areas critical for VT maintenance within the dense scar. This is a stepwise approach that starts with the identification of the potential critical isthmus within the dense scar that is related to the patient’s clinical and/or induced VTs based on conventional criteria including voltage channels; sites with LPs; sites with good pacemaps; and the existence of long stimulus to QRS intervals, isthmus sites defined by entrainment mapping, and sites of VT termination with ablation. Therefore, this approach acts as a combined approach between conventional and substrate-based approaches. These areas are typically within areas of dense scar (< 0.5 mV). Once identified, the critical area is targeted with contiguous ablation lesions either completely surrounding the region of interest or by using anatomic anchors to minimize the amount of ablation necessary. The authors demonstrated that, after a mean follow-up of 18 months ± nine months, no VT recurrence was observed in 38 (86%) of 44 patients.^79^

#### Scar dechanneling

This substrate ablation approach, which targets channels within the abnormal substrate, was originally described by Soejima et al.^45^ and Arenal et al.^80^ Although, in all studies, the concept of scar dechanneling encompasses targeting the VT channels, the identification of the channels differs in terms of technique. In the study by Soejima et al.,^45^ channels were identified within the low-voltage area using high-output pacing (10 mA, with pulse width of 2 ms). Electrically unexcitable scar was defined as a loss of capture at high-output pacing and marked on the voltage maps. Conversely, Arenal et al.^80^ were able to visualize channels after adjusting voltage cutoffs on EAM. More recently, Tung et al.^81^ and Berruezo et al.^82^ described an approach that targets interconnected activation channels within the abnormal substrate, adopting clear endpoints with clinical follow-up. The method involved high-density mapping of the channels of activation of LPs. Once a specific sequence of LP activation has been identified, focal ablation of the earliest LP is delivered with the end goal of eliminating a consecutive series of LPs. Tung et al.^81^ demonstrated a rate of freedom from VT recurrence of 86% (18/21) during a median follow-up of 11 months (range: 6–18 months), while Berruezo et al.^82^ noted that, during a median follow-up of 21 months (range: 11–29 months), the rate of freedom of VT recurrence was 80% (80/101). In addition, Andreu et al.^83^ recently demonstrated scar dechanneling by using CMR imaging in conjunction with EAM, which showed corridors formed by conducting channel points in the scar tissue. In that study, the rate of VT freedom during a mean follow-up of 20 months ± 19 months was 81.5% (44/54).^83^

### Frequency analysis mapping

High-frequency electrogram components are more often associated with critical sites of reentry as compared with low-frequency, large-amplitude components. Several studies have demonstrated that the frequency analysis of electrograms may aid with substrate identification^84–86^; however, this analysis is still only available as an offline tool and the feasibility of an automated real-time tool needs to be further investigated.

### Use of imaging to identify substrate

**Figure 3:**
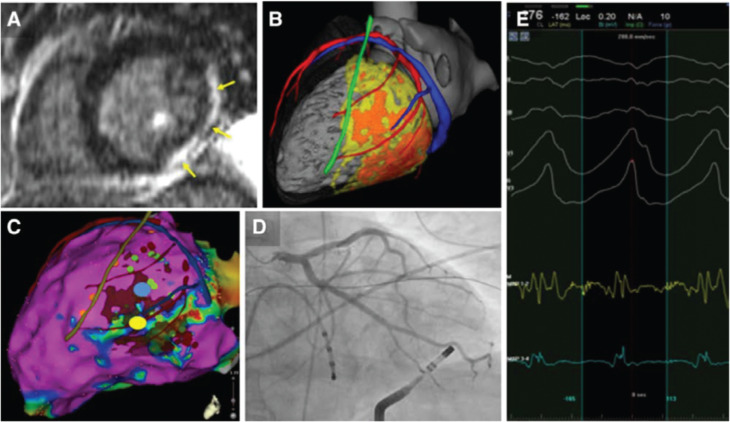
**A:** Lateral and inferior LV scar on CMR (arrows). **B:** Patient-specific 3D model built from merged computed tomography (CT) (anatomy) and magnetic resonance imaging (scar) data. Cardiac chambers (gray), coronary arteries, veins (in red and blue, respectively), and left phrenic nerve (green) as segmented from CT and dense scar and gray zone (in orange and yellow, respectively) as segmented from magnetic resonance imaging. **C:** Epicardial bipolar voltage map with merged imaging model. Voltage mapping (color-coded from purple to red) underestimates the substrate extent as compared with imaging. Fractionated and LPs (green dots) are identified in normal voltage areas. Middiastolic potentials (yellow and blue signals in **E**) are recorded during VT on an epicardial lateral LV site (yellow dot in **C**). This potential target for epicardial ablation is far enough from the left phrenic nerve path derived from imaging (green line in **C**), which accurately matches sites of phrenic capture (orange dots in **C**). However, CT demonstrates the proximity of this site to a marginal branch of the circumflex artery on the registered imaging model. **D:** Confirmatory coronary angiography demonstrates contact between the tip of the ablation catheter and the coronary artery. Ablation was thus performed on a different site of the VT isthmus (blue dot in **C**), resulting in successful VT termination. Reproduced with permission from Mahida S, Sacher F, Dubois R, et al. Cardiac imaging in patients with ventricular tachycardia. *Circulation*. 2017;136(25):2491–2507.

Cardiac imaging may play an important role in the preprocedural assessment of cardiac anatomy and myocardial scar as well as in the intraprocedural integration of the structural VT substrate.^28^ Cardiac imaging has been mainly used as an adjunct either offline or online (real-time image integration)^27,83,87,88^ to support the localization of scar in 3D mapping systems during substrate mapping and ablation **(Figure 3)**. Cardiac imaging has several advantages, as follows: (1) it may provide precise anatomical information including endocardial/intramural/epicardial scar location, while EAM systems can only provide derived 3D reconstructions from catheter–electrode contact at the myocardial surface; (2) there is no possibility of inaccuracy due to extrapolation, lack of catheter contact, confounding effects of far-field electrograms, or the influence of wavefront activations; (3) it provides information about adjacent anatomical structures, which may affect mapping and ablation, such as papillary muscles, coronary arteries, phrenic nerves, and epicardial fat. However, there are also limitations in imaging techniques in terms of feasibility (eg, magnetic resonance imaging in some patients with old ICDs, MDCT in patients with severe chronic kidney failure), the availability of images with 3D mapping systems, and registration issues.

Recently, in an attempt to refine targeted VT ablation strategies further, several studies have focused on identifying specific scar regions that harbor critical VT isthmuses.^29,67,89^ At this time, scar regions with increased transmurality, scar border zones, and regions at the scar-core–border-zone transition point have been identified as potential targets.^29,90^ CMR has been widely used in this regard, and several studies have shown good correlation with EAM^30,31,62,83,88^ and a positive clinical impact.^27,52,87^ Further potential benefits of real-time CMR guidance^91–93^ could include improved procedural supervision without exposure to radiation/contact EAM as well as improved substrate detection and lesion visualization according to CMR-defined endpoints. However, CMR may be unavailable, contraindicated, or of suboptimal quality because of ICD-related artifacts, and MDCT represents a valuable alternative for imaging integration. MDCT has been used in combination with EAM to accurately identify dense scar and border-zone regions with significantly higher special resolution^29,68^ as compared with CMR and with high clinical effectiveness.^27^ Studies have mostly included patients with ICM,^29^ while the correlation in patients with NICM is less robust.^67,68,87,89^ A further advantage of MDCT is in the definition of high-resolution cardiac anatomy.^47,94^ CMR and MDCT may visualize potential isthmuses as VT substrate; however, a certain proportion of circuits are at least partially functional^95^ and the registration needs to be accurate and reproducible.

Other modalities such as intracardiac echocardiography^96,97^ and nuclear imaging^98,99^ may also help to identify substrate. In addition, electrocardiographic imaging incorporated with CMR or MDCT has the potential to identify VT isthmuses noninvasively.^100^ Furthermore, an entirely noninvasive approach that combines anatomical imaging with electrocardiographic imaging and noninvasive cardiac radiotherapy for ablation has been reported, which represents another intriguing strategy that employs cardiac imaging.^101^

### Clinical outcomes of substrate-based ablation

Overall, the clinical outcome of substrate-based ablation is a VT recurrence-free rate of approximately 54% to 91% in mid- to long-term follow-up **(Table 1)**. As described above, the success rates, however, vary widely with different strategies and across studies. We have also compared clinical outcomes between substrate- and nonsubstrate-based ablation **(Table 2)**. Di Biase et al.^11^ demonstrated a superior VT-free survival rate at 12 months in conjunction with extensive scar homogenization in patients with ICM in a randomized trial [ie, the Ablation of Clinical VT versus Addition of Substrate Ablation on the Long-term Success Rate of VT Ablation (VISTA) trial], while Fernández-Armenta et al.^102^ also conducted a randomized study comparing substrate-based ablation to conventional ablation and demonstrated comparable VT-free survival rates between the two strategies. A recent meta-analysis has also revealed similar acute procedural efficacy, complication, VT recurrence, and mortality rates while comparing a predominantly substrate-based ablation strategy to a strategy guided by activation and entrainment mapping of inducible and hemodynamically tolerated VTs.^103^ A separate meta-analysis^104^ also showed a significantly lower risk of the composite primary outcome of long-term VA recurrence and all-cause mortality among those undergoing substrate modification in comparison with standard ablation in a cohort of mostly patients with ICM. Furthermore, in this study, long-term success was improved when performing complete substrate modification.^104^ Hence, substrate ablation may be superior to a conventional strategy in terms of VT recurrence when extensive substrate ablation is performed.^11^

However, despite the substantial progress that has been made in the use of cardiac imaging to guide VT ablation, there is insufficient evidence at present to suggest that the use of imaging can add value to clinical outcomes. Although observational, nonrandomized studies suggest that image integration may have an impact on procedural outcomes,^27,83,87,88^ well-designed, prospective randomized studies are needed to assess the true impact of image integration as well as to evaluate the potential mechanism(s) of any benefit.

## Conclusion

Several substrate-based ablation strategies have been developed, which include extensive or less-extensive ablation lesions according to the preset mapping and endpoints of the procedure. Although multipolar mapping catheters with smaller and more-narrowly-spaced bipolar electrodes are now widely used, most currently available studies use data acquired by way of ablation catheters. Advances in cardiac imaging may be helpful in providing refined anatomical substrate details.

At this time, clinical outcomes of substrate-based ablation are at least comparable with and possibly superior to conventional VT ablation. The further development of mapping technologies, cardiac imaging, and novel modalities and the incorporation of these modalities in delineating VT substrate may additionally improve the clinical outcomes of substrate-based ablation.

## Figures and Tables

**Table 1:** Studies Investigating Different Substrate Ablation Strategies for VT

Ablation Method	Study	Patients	Age	LVEF	Endpoint(s) [% of Patients Who Met the Endpoint(s)]	Epicardial and Endocardial Ablation/Mapping	3D Mapping System
Linear ablation	Marchlinski et al. 2000^10^	9 ICM and 7 NICM	58 ± 16 years	32% ± 15%	Noninducibility (47%)	0/0	CARTO^®^ in 13 patients; none in 3 patients
	Soejima et al. 2001^72^	40 ICM	67 ± 11 years	28.9%	Noninducibility (58%)	0/0	CARTO^®^ in 40 patients
LP ablation	Volkmer et al. 2006^16^	25 ICM	65 ± 8 years	30% ± 8%	Elimination of LPs and noninducibility (81%)	0/0	CARTO^®^ in 25 patients
	Arenal et al. 2003^73^	21 ICM, 2 NICM, and 1 TOF	66 ± 9 years	30% ± 9%	Elimination of LPs and noninducibility (88%)	0/0	CARTO in 22 patients; none in 2 patients
	Nogami et al. 2008^74^	18 ARVC	48 ± 11 years	N/A	Change of isolated delayed component and noninducibility (33%)	0/0	CARTO^®^ in 18 patients
	Garcia et al. 2009^75^	13 ARVC	43 ± 15 years	N/A	Elimination of LPs and noninducibility (85%)	13/13 (1 underwent both types of mapping and epicardial ablation but not endocardial ablation)	CARTO^®^ in 13 patients
	Bai et al. 2011^76^	26 ARVC	37 ± 11 years	53% ± 10%	Elimination of LPs and noninducibility (100%)	26/26	CARTO^®^ in 26 patients
	Vergara et al. 2012^77^	36 ICM and 14 NICM	66 ± 10 years	32% ± 9% for ICM; 36% ± 10% for NICM	Elimination of LPs (84%)	21/21 (3 underwent both types of mapping and epicardial ablation but not endocardial ablation)	CARTO^®^/NavX™ (number unknown)
	Arenal et al. 2013^78^	59 ICM	67 ± 9 years	30% ± 11%	Elimination of LPs (78%)	0/0	CARTO^®^ in 59 patients
LAVA	Jaïs et al. 2012^19^	56 ICM 14 NICM	67 ± 11 years	35% ± 10%	Elimination of LAVA (70%) and noninducibility	17/21	CARTO^®^/NavX™ (number unknown)
	Wolf et al. 2018^20^	159 ICM	65 ± 11 years	34% ± 11%	Elimination of LAVA (64%) and noninducibility	27/46	CARTO^®^/NavX™/Rhythmia™ in a total of 119 patients; none in 40 patients
Scar homogenization	Di Biase et al. 2015^11^	58 ICM	67 ± 9 years	32% ± 10%	Elimination of any abnormal potential ± loss of pacing capture (NA).	NA	CARTO^®^ in 58 patients
	Di Biase et al. 2012^17^	43 ICM	62 ± 8 years	24% ± 8%	Elimination of any abnormal potential ± loss of pacing capture (NA).	14/43	CARTO^®^ in 43 patients
Scar dechanneling	Berruezo et al. 2012^38^	11 ARVC	42 ± 13 years	55% ± 7%	Elimination of LP channels (NA)	11/11	CARTO^®^ in 11 patients
	Tung et al. 2013^81^	15 ICM, 2 NICM, 2 ARVC, 1 sarcoid, 1 noncompaction, and 1 Chagas	63 (52–70) years	25% (25%–30%)	Change or elimination of LPs, failure to capture ± impedance drop of ; 10 Ω plus noninducibility (84%)	9/9	CARTO^®^ in 11 patients patients/NavX™ in 10 patients
	Berruezo et al. 2015^82^	75 ICM and 26 NICM	65 ± 12 years	36% ± 13%	Elimination of LP channels (84%)	N/A/27	CARTO^®^ in 96 patients/NavX™ in 5 patients
	Fernández-Armenta et al. 2016^102^	19 ICM and 5 NICM	66 ± 11 years	36% ± 14%	Elimination of delayed components (87.5%)	5/5 (1 underwent epicardial ablation and mapping but not endocardial ablation or mapping)	CARTO/NavX (n = N/A)
Core isolation	Tzou et al. 2015^79^	32 ICM and 12 NICM	63 ± 14 years	31% ± 13%	Isolation with exit block (84%)	5/6	CARTO^®^ in 44 patients
MRI-based scar dechanneling	Andreu et al. 2017^83^	37 ICM and 17 NICM	64 ± 11 years	38% ± 12%	Elimination of LP channels detected on EAM guided by CMR (84%)	18/18	CARTO^®^ in 54 patients

ARVC: arrhythmogenic right ventricular cardiomyopathy; CMR: cardiac magnetic resonance imaging; EAM: electroanatomical mapping; Epi: epicardial; ICM: ischemic cardiomyopathy; LAVA: local abnormal ventricular activity; LP: late potential; LVEF: left ventricular ejection fraction; LP: late potential; MRI: magnetic resonance imaging; N/A: not available; NICM: nonischemic cardiomyopathy; noncompaction: noncompaction cardiomyopathy; RF: radiofrequency; sarcoid: cardiac sarcoidosis; TOF: tetralogy of Fallot; VT: ventricular tachycardia. CARTO^®^ is the property of Biosense Webster, Diamond Bar, CA, USA. NavX™ is the property of Abbott Laboratories, Chicago, IL, USA. Rhythmia™ is the property of Boston Scientific, Natick, MA, USA.

**Table 1:** Studies Investigating Different Substrate Ablation Strategies for VT (continued)

Ablation Method	Study	Mapping Catheter	Voltage Map Cutoff	Procedure Time	Ablation Catheter	RF Time or No. of Lesions
Linear ablation	Marchlinski et al. 2000^10^	Ab cath	0.5–1.5 mV	9.4 hours	Nonirrigated	59 ± 34 lesions
	Soejima et al. 2001^72^	Ab cath	0.5–1.5 mV	7.4 hours	Nonirrigated/open irrigated/closed irrigated	21 ± 10 lesions
LP ablation	Volkmer et al. 2006^16^	Ab cath	0.5–1.5 mV	3.9 ± 1.1 hours	Noirrigated	11 ± 8 lesions
	Arenal et al. 2003^73^	Ab cath	0.5–1.5 mV	7.9 ± 2.1 hours	Nonirrigated/open irrigated	14 ± 6 lesions
	Nogami et al. 2008^74^	Ab cath	0.1–1.5 mV	3.6 ± 1.1 hours	Nonirrigated	17 ± 10 lesions
	Garcia et al. 2009^75^	Ab cath	0.5–1.5 mV (endo)/ 0.5–1.0 mV (epi)	N/A	Nonirrigated/open irrigated	35 ± 26 lesions (endo)/ 37 ± 21 lesions (epi)
	Bai et al. 2011^76^	Ab cath	0.5–1.5 mV (endo)/ 0.5–1.5 mV (epi)	5.3 ± 1.2 hours	Open irrigated	26 ± 14 minutes
	Vergara et al. 2012^77^	Ab cath/Livewire™/AFocus™ II	0.5–1.5 mV (endo)/ 0.5–1.0 mV (epi)	NA	Open irrigated	N/A
	Arenal et al. 2013^78^	Ab cath	0.1–1.5 mV	2.9 ± 0.8 hours	Open irrigated	11 ± 5 minutes
LAVA	Jaïs et al. 2012^19^	Ab cath/PentaRay^®^	0.5–1.5 mV (endo)/ 0.5–1.0 mV (epi)	2.5 ± 1.2 hours	Open irrigated	23 ± 11 minutes
	Wolf et al. 2018^20^	Multielectrode catheter in 89 patients/Ab cath in 70 patients	0.5–1.5 mV (endo)/ 0.5–1.0 mV (epi); 0.2–0.8 mV was used in 6 patients with Rhythmia™	4.1 ± 1.3 hours	Open irrigated	36 ± 20 minutes
Scar homogenization	Di Biase et al. 2015^11^	Ab cath	0.5–1.5 mV (endo)/ 0.5–1.5 mV (epi)	4.2 ± 1.3 hours	Open irrigated	68 ± 21 minutes
	Di Biase et al. 2012^17^	Ab cath	0.5–1.5 mV (endo)/ 0.5–1.5 mV (epi)	4.8 ± 1.5 hours	Open irrigated	74 ± 21 minutes
Scar dechanneling	Berruezo et al. 2012^38^	Ab cath	0.5–1.5 mV (endo)/ 0.5–1.5mV (epi)	3.0 ± 1.0 hours	Open irrigated	6.3 (4–8.7) lesions
	Tung et al. 2013^81^	DecaNav^®^ in 3 patients/Livewire™ in 16 patients/Constellation™ in 2 patients	0.5–1.5 mV (endo)/ 0.5–1.5 mV (epi)	N/A	Open irrigated (including Thermocool^®^ SF)/closed irrigated	7 (4–14) lesions
	Berruezo et al. 2015^82^	NA	0.5–1.5 mV (endo)/ 0.5–1.5 mV (epi)	3.8 ± 1.1 hours	Open irrigated	28 ± 16 minutes
	Fernández-Armenta et al. 2016^102^	Ab cath	0.5–1.5 mV (endo)/ 0.5–1.5 mV (epi)	3.5 ± 1.1 hours	Open irrigated	23 ± 14 minutes
Core isolation	Tzou et al. 2015^79^	Ab cath	0.5–1.5 mV (endo)/ 0.5–1.0 mV (epi)	5.4 ± 2.0 hours	Open irrigated (including Thermocool^®^ SF)	111 ± 91 lesions
MRI-based scar dechanneling	Andreu et al. 2017^83^	Ab cath	0.5–1.5 mV (endo)/ 0.5–1.5 mV (epi)	3.8 ± 1.1 hours	Open irrigated	19 ± 12 minutes

Ab cath: ablation catheter; LAVA: local abnormal ventricular activity; LP: late potential; MRI: magnetic resonance imaging; No.: number; RF: radiofrequency. Livewire™ and AFocus™ II are the property of Abbott Laboratories, Chicago, IL, USA. DecaNav^®^, PentaRay^®^, and Thermocool^®^ SF are the property of Biosense Webster, Diamond Bar, CA, USA. Constellation™ is the property of Boston Scientific, Natick, MA, USA.

**Table 1:** Studies Investigating Different Substrate Ablation Strategies for VT (continued)

Ablation Method	Study	Complication(s)	Follow-up (Mean or Median)	VT Recurrence (%)	Mortality (%)
Linear ablation	Marchlinski et al. 2000^10^	1 (stroke)	8 (3–36) months	4 (25%)	N/A
	Soejima et al. 2001^72^	4 (1 iliac artery dissection, 1 femoral artery pseudoaneurysm, 1 embolism to lower leg, 1 retroperitoneal hematoma)	12 ± 6 months	15 (37.5%)	9 (22.5%); 5 noncardiac-related and unrelated to the procedure, 3 due to cardiac failure, 1 sudden death
LP ablation	Volkmer et al. 2006^16^	0	9 ± 4 months	5 (21%)	1 (4.2%) noncardiac cause
	Arenal et al. 2003^73^	0	26 ± 14 months	7 (29%)	2 (8%); 1 tamponade in hospital, 1 nonarrhythmogenic death
	Nogami et al. 2008^74^	N/A	61 ± 38 months	6 (33%)	3 (17%); 2 heart failure, 1 malignancy
	Garcia et al. 2009^75^	0	18 ± 13 months	3 (23%)	N/A
	Bai et al. 2011^76^	1 (groin hematoma)	39 ± 4 months	4 (15%)	0 (0%)
	Vergara et al. 2012^77^	N/A	13 ± 4 months	10 (20%)	1 (2%) heart failure
	Arenal et al. 2013^78^	0	39 ± 21 months	25 (42%)	13 (22%); 6 heart failure, 3 recurrent incessant VT, 1 sudden cardiac death, 3 noncardiac-related
LAVA	Jaïs et al. 2012^19^	2 (1 cardiac tamponade, 1 RV perforation)	22 (14–27) months	32 (46%)	13 (19%); 2 died within 24 hours from ablation, 1 PEA, 2 heart failure, 1 sudden death, 1 VT storm, 6 noncardiac-related
	Wolf et al. 2018^20^	12 (9 epicardial bleeding, 2 complete AV block, 1 acute heart failure)	47 (33–82) months	71(45%)	40 (25%); 3 arrhythmia-related, 20 heart failure, 17 noncardiac-related
Scar homogenization	Di Biase et al. 2015^11^	3 (pericardial effusion)	12 months	9 (15.5%)	5 (8.6%); 3 nonarrhythmic cardiac-related 2 noncardiac-related
	Di Biase et al. 2012^17^	1 (groin hematoma)	21 (19–25) months	8 (19%)	1 (2%) noncardiac-related
Scar dechanneling	Berruezo et al. 2012^38^	1 (RV puncture)	11 (6–24) months	1 (9%)	0 (0%)
	Tung et al. 2013^81^	N/A	11 (6–18) months	3 (14%)	N/A
	Berruezo et al. 2015^82^	7 (2 tamponade, 2 complete AV block, 2 pericardial effusion, 1 TIA, 1 PN palsy)	21 (11–29) months	20 (20%)	9 (8.9%); 4 heart failure, 1 sudden cardiac death, 1 arrhythmic storm, 2 noncardiac-related, 1 unknown
	Fernández-Armenta et al. 2016^102^	0	22 ± 14 months	10 (41.7%)	N/A
Core isolation	Tzou et al. 2015^79^	2 (1 arterial pseudoaneurysm, 1 transient hypotension)	18 ± 9 months	6 (14%)	0 (0%)
MRI-based scar dechanneling	Andreu et al. 2017^83^	N/A	20 ± 19 months	10 (18.5%)	0 (0%)

AV: atrioventricular; CHF: congestive heart failure; DVT: deep vein thrombosis; LP: late potential; LAVA: local abnormal ventricular activity; MRI: magnetic resonance imaging; PN: phrenic nerve; RV: right ventricle/ventricular; TIA: transient ischemic attack.

**Table 2:** Summary of Studies Comparing Outcomes between Substrate and Conventional Ablation Strategies

Study	Design	Patients	Age (Mean or Median)	LVEF (%)	Strategy (Substrate Ablation)	Endpoint(s)	Follow-up (Mean or Median)	No. of Sub	Sub VT	Sub Complications	Sub Cardiac Death	Sub All-cause Death	No. of Controls	Non-sub VT	Non-sub Complications	Non-sub Cardiac Death	Non-sub All Death	Results
Di Biase et al. 2015^11^	Multicenter, prospective randomized study	118 ICM	67 ± 9 years (sub) and 65 ± 12 years (control)	32% ± 10% (sub) and 33% ± 14% (control)	Scar homogenization	Elimination of any abnormal potential ± loss of pacing capture (NA)	12 months	58	9 (16%)	3 (5%)	3 (5%)	5 (9%)	60	29 (48%)	3 (5%)	5 (8%)	9 (15%)	Substrate ablation better in ventricular arrhythmia recurrence during one-year follow-up (log-rank < 0.001); mortality or complication was comparable (p = 0.54 and p = 0.61)
Bunch et al. 2012^13^	Multicenter, retrospective case-control study	31 (20 ICM and 11 NICM)	62.5 years (sub) and 59.7 years (control)	25% (sub) and 20% (control)	LP ablation/linear ablation	Noninducibility	9 ± 3 months	18	8 (44%)	3 (17%)	4 (22%)	4 (22%)	13	4 (31%)	4 (31%)	1 (8%)	1 (8%)	No statistical difference in VT recurrence, complication, cardiac death, or all-cause death
Makimoto et al. 2015^14^	Single-center, retrospective case-control study	85 (34 ARVC, 16 ICM, 14 DCM, 1 HCM, 2 D-HCM, 11 sarcoidosis, and 6 congenital)	53.1 ± 16.2 years	51.7% ± 16.4%	LP ablation/linear ablation	Elimination of fractionated or isolated delayed potentials, confirmation of a linear lesion blockade PVC elimination, noninducibility	61 ± 40 months	50	15 (30%)	0 (0%)	NA	NA	35	15 (43%)	0 (0%)	3 (9%)	NA	No statistical difference in VT recurrence, heart failure, or death during five years of follow-up
Ventura et al. 2007^15^	Single-center, prospective cohort study	30 ICM	65 ± 7 years	32% ± 6%	Pacemap-based ablation	Local pacing capture loss and noninducibility	14 ± 6 months	14	6 (43%)	0 (0%)	NA	NA	16	4 (25%)	1 (6%)	NA	NA	No statistical difference in VT recurrence
Volkmer et al. 2006^16^	Single-center, retrospective case-control study	47 ICM	65 ± 8 years	30% ± 7% (sub) and 30% ± 7% (control)	LP ablation	Elimination of LPs related to the VT and noninducibility	24 ± 12 (sub) months, 26 ± 14 (control) months	25	7 (28%)	3 (12%)	3 (12%)	3 (12%)	22	6 (27%)	2 (9%)	3 (14%)	4 (18%)	No statistical difference in VT recurrence or death
Di Biase et al. 2012^17^	Multicenter, prospective cohort study	92 ICM	62 ± 13 years	27% ± 5% (sub) and 24% ± 8% (control)	Scar homogenization	Elimination of any abnormal potential ± loss of pacing capture (NA)	25 ± 10 months	43	8 (19%)	0 (0%)	0 (0%)	0 (0%)	49	23 (47%)	0 (0%)	0 (0%)	0 (0%)	Substrate ablation better in ventricular arrhythmia recurrence during follow-up (log-rank = 0.006)
Fernández-Armenta et al.^102^	Single-center, prospective randomized study	48 (37 ICM and 11 NICM)	66 ± 11 years (sub) and 69 ± 8 years (control)	36% ± 14% (sub) and 36% ± 11% (control)	Scar dechanneling	Elimination of delayed components	22 ± 14 months	24	10 (42%)	0 (0%)	0 (0%)	NA	24	8 (33%)	4 (17%)	0 (0%)	NA	No statistical difference in VT recurrence during follow-up

ARVC: arrhythmogenic right ventricular cardiomyopathy; DCM: dilated cardiomyopathy; D-HCM: hypertrophic cardiomyopathy dilated phase; HCM: hypertrophic cardiomyopathy; ICM: ischemic cardiomyopathy; LP: late potential; LVEF: left ventricular ejection fraction; N/A: not available; NICM: nonischemic cardiomyopathy; No.: number; sub: substrate ablation strategy; VT: ventricular tachycardia recurrence.
